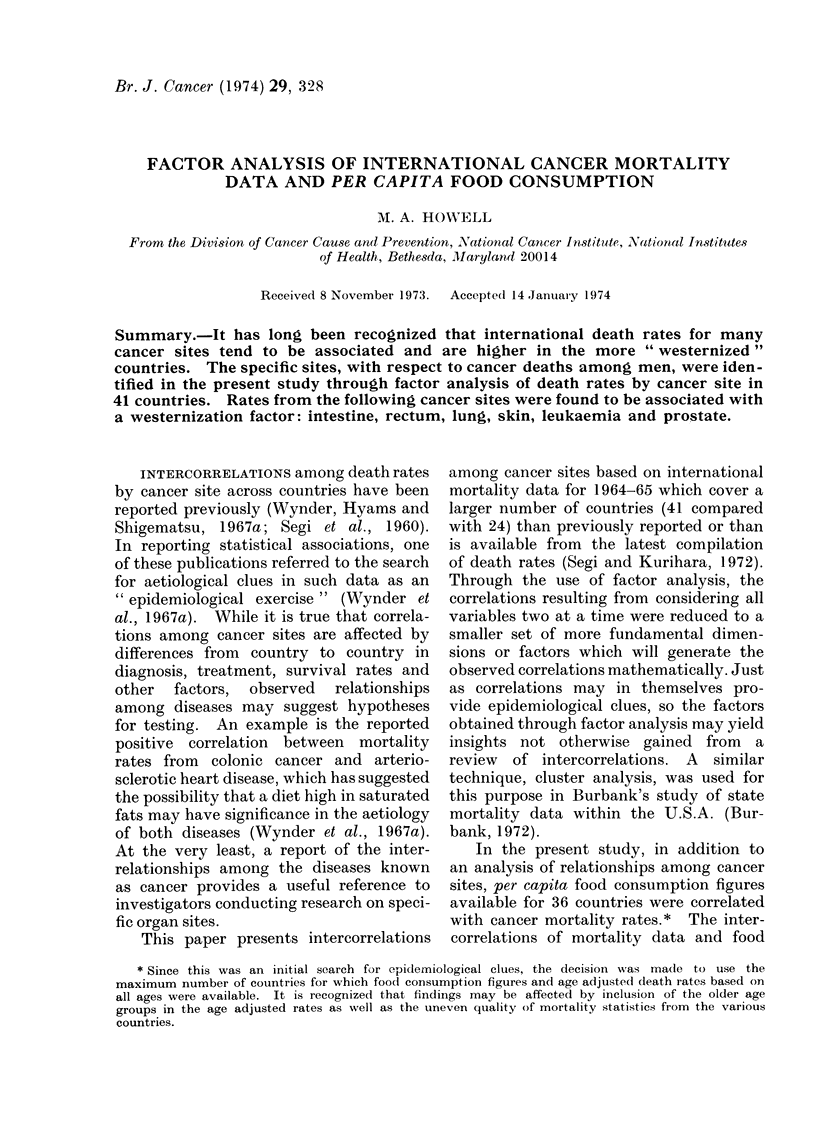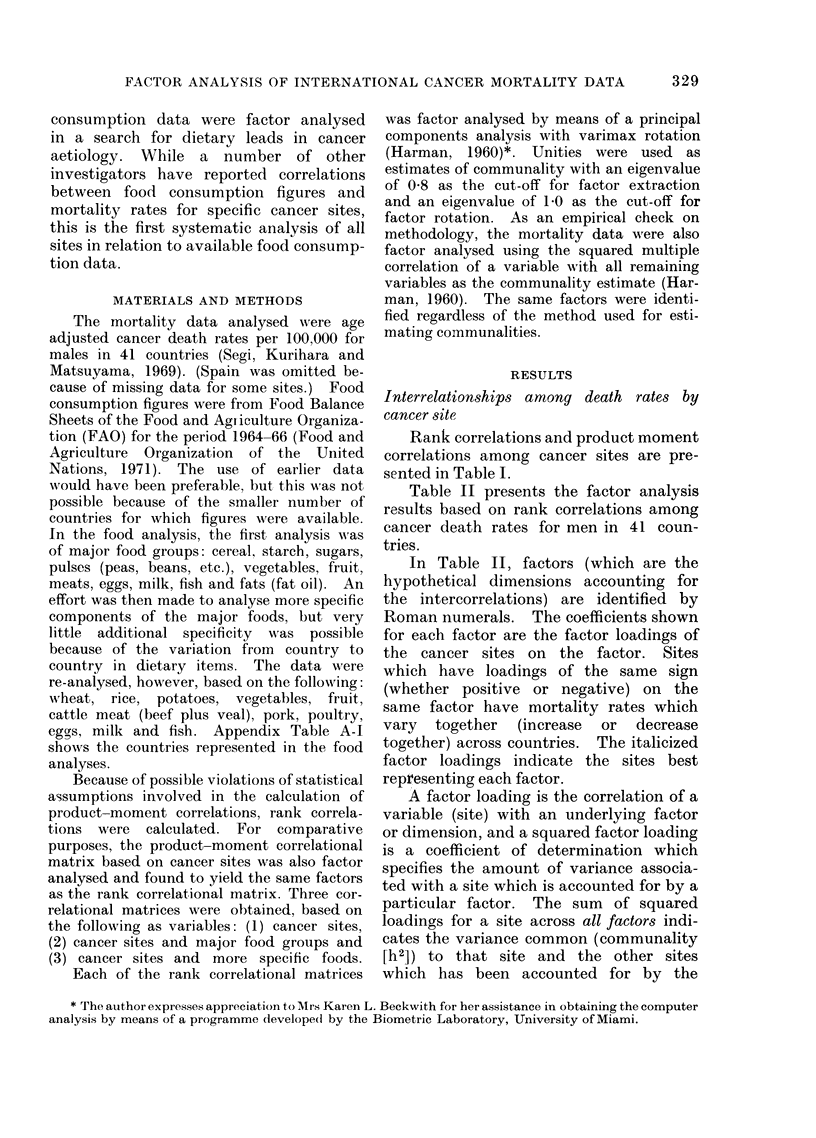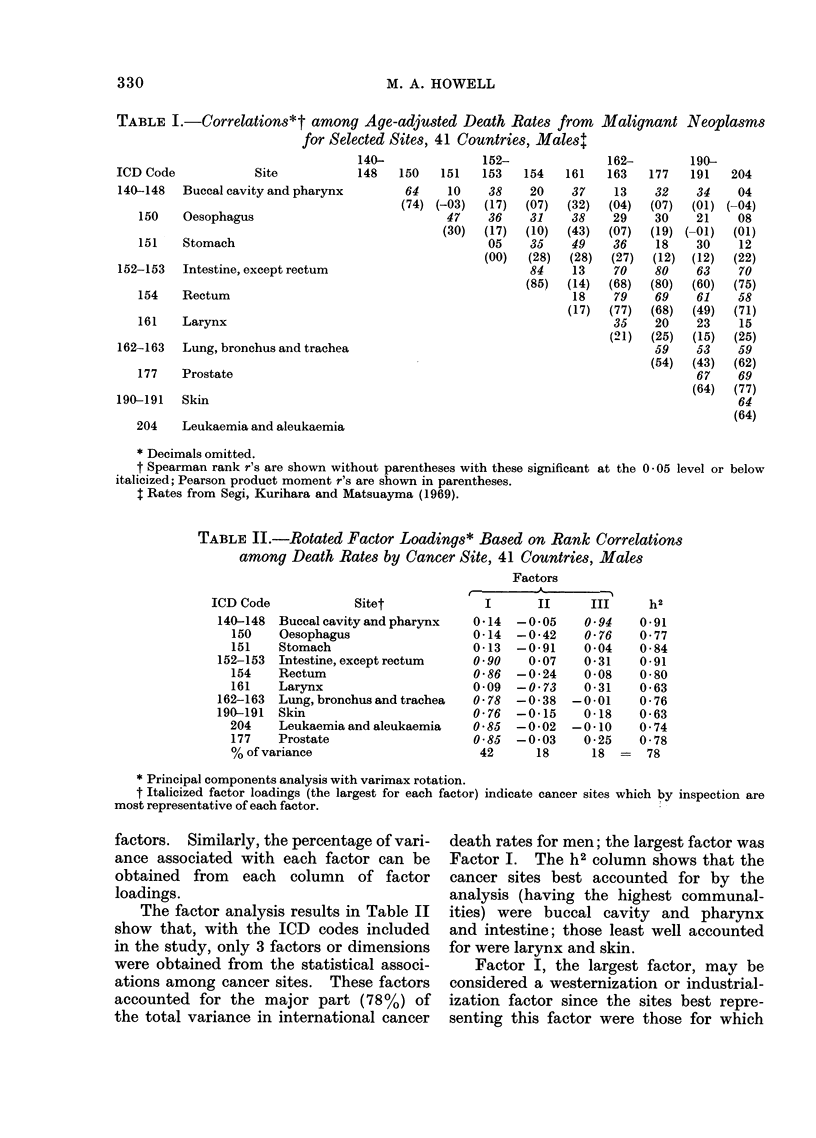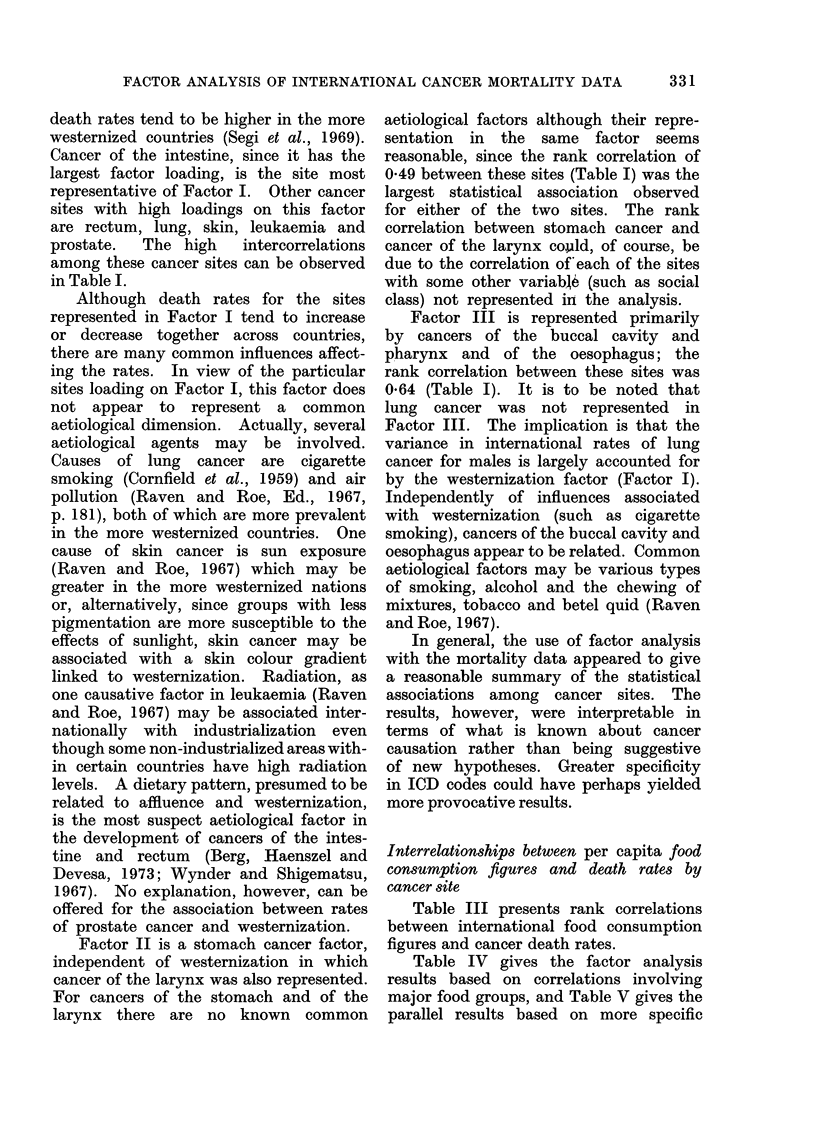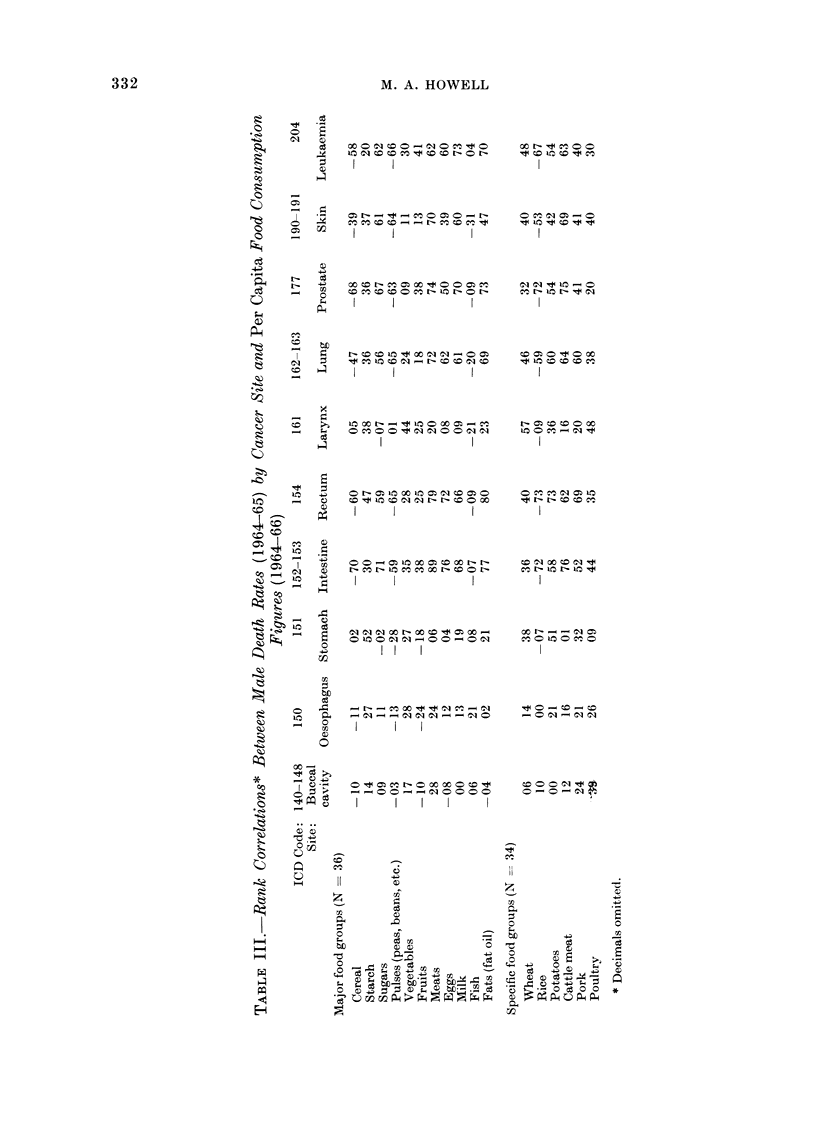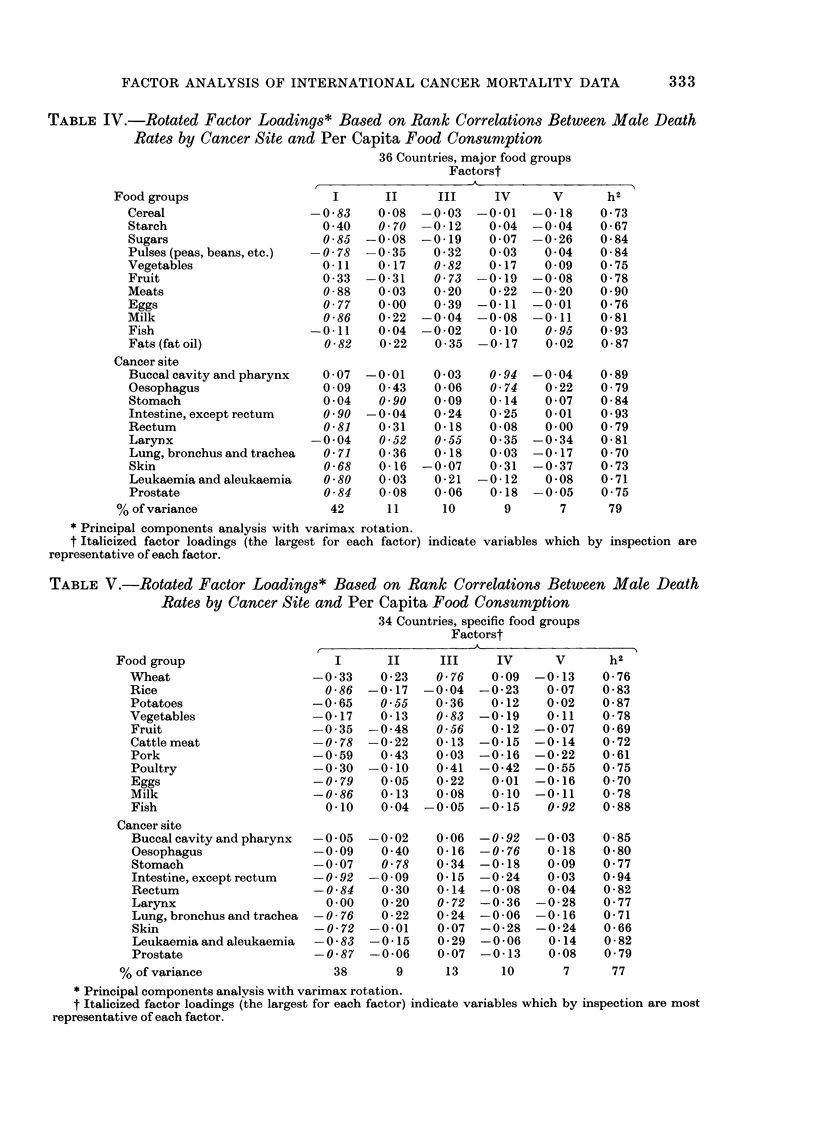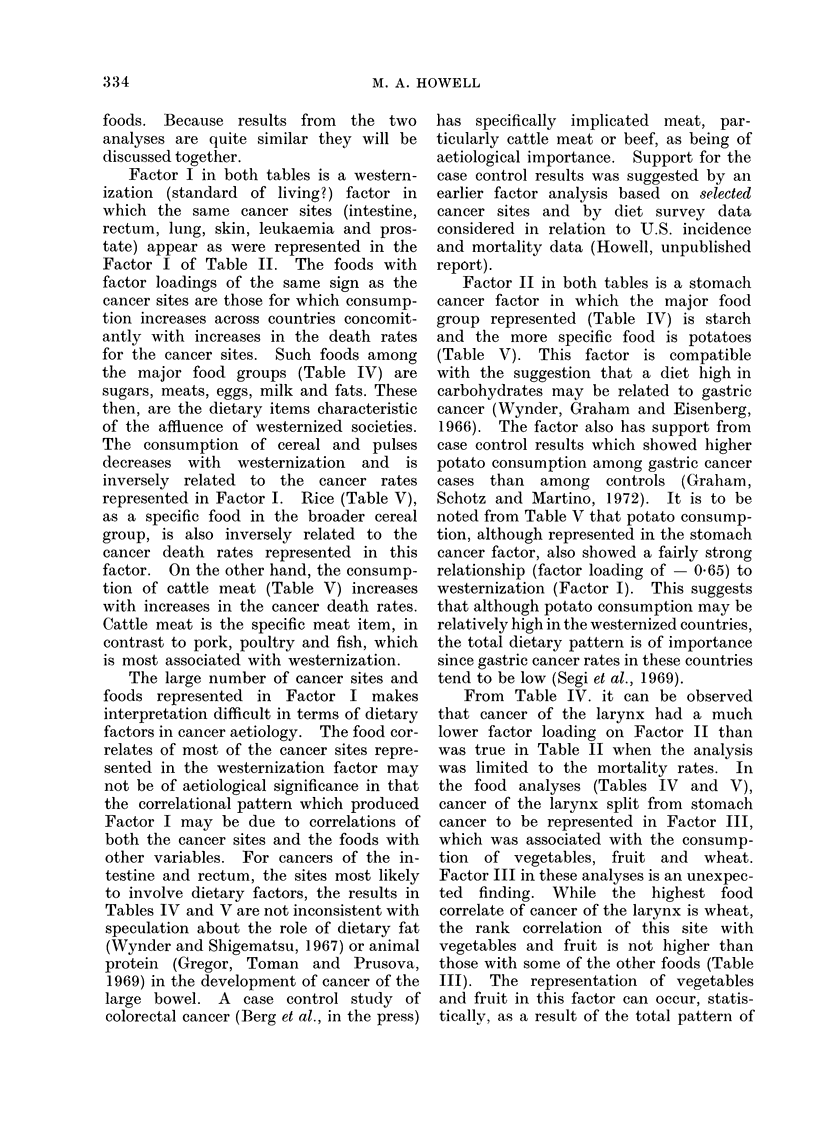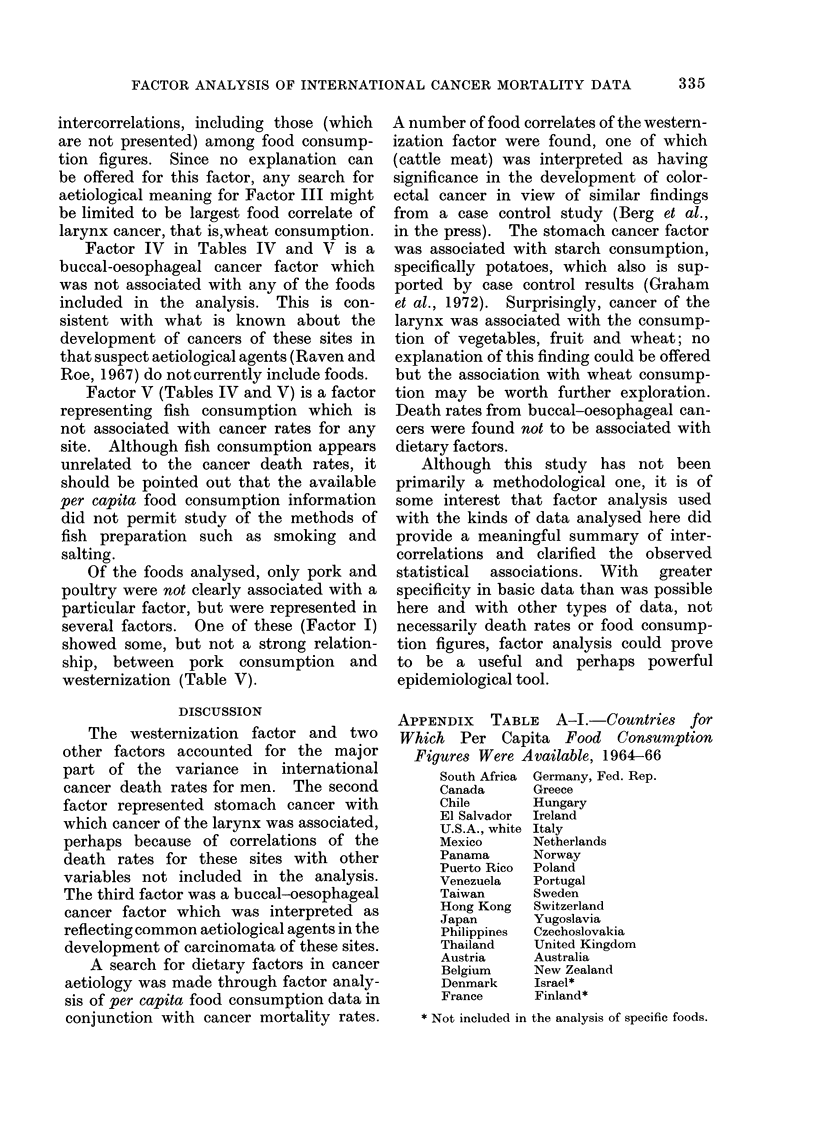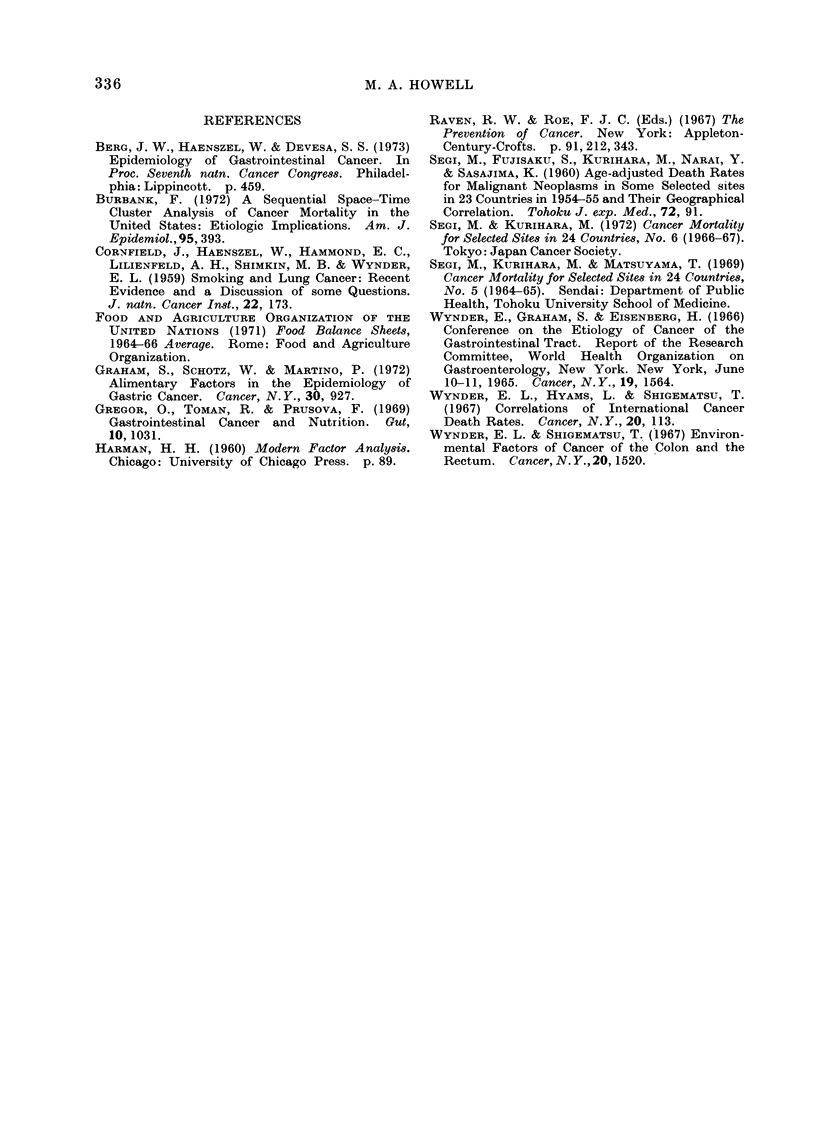# Factor Analysis of International Cancer Mortality Data and per capita Food Consumption

**DOI:** 10.1038/bjc.1974.75

**Published:** 1974-04

**Authors:** M. A. Howell

## Abstract

It has long been recognized that international death rates for many cancer sites tend to be associated and are higher in the more “westernized” countries. The specific sites, with respect to cancer deaths among men, were identified in the present study through factor analysis of death rates by cancer site in 41 countries. Rates from the following cancer sites were found to be associated with a westernization factor: intestine, rectum, lung, skin, leukaemia and prostate.


*Br. J. Cancer* (1974) **29**, 328

### FACTOR ANALYSIS OF INTERNATIONAL CANCER MORTALITY DATA AND *PER CAPITA* FOOD CONSUMPTION

M. A. HOWELL

*From the Division of Cancer Cause and Prevention, National Cancer Institute, National Institutes of Health, Bethesda, Maryland* 20014

Received 8 November 1973. Accepted 14 January 1974

**Summary.—It has long been recognized that international death rates for many cancer sites tend to be associated and are higher in the more "westernized" countries. The specific sites, with respect to cancer deaths among men, were identified in the present study through factor analysis of death rates by cancer site in 41 countries. Rates from the following cancer sites were found to be associated with a westernization factor: intestine, rectum, lung, skin, leukaemia and prostate.**

INTERCORRELATIONS among death rates by cancer site across countries have been reported previously (Wynder, Hyams and Shigematsu, 1967*a*; Segi *et al.*, 1960). In reporting statistical associations, one of these publications referred to the search for aetiological clues in such data as an "epidemiological exercise" (Wynder *et al.*, 1967*a*). While it is true that correlations among cancer sites are affected by differences from country to country in diagnosis, treatment, survival rates and other factors, observed relationships among diseases may suggest hypotheses for testing. An example is the reported positive correlation between mortality rates from colonic cancer and arteriosclerotic heart disease, which has suggested the possibility that a diet high in saturated fats may have significance in the aetiology of both diseases (Wynder *et al.*, 1967*a*). At the very least, a report of the interrelationships among the diseases known as cancer provides a useful reference to investigators conducting research on specific organ sites.

This paper presents intercorrelations among cancer sites based on international mortality data for 1964–65 which cover a larger number of countries (41 compared with 24) than previously reported or than is available from the latest compilation of death rates (Segi and Kurihara, 1972). Through the use of factor analysis, the correlations resulting from considering all variables two at a time were reduced to a smaller set of more fundamental dimensions or factors which will generate the observed correlations mathematically. Just as correlations may in themselves provide epidemiological clues, so the factors obtained through factor analysis may yield insights not otherwise gained from a review of intercorrelations. A similar technique, cluster analysis, was used for this purpose in Burbank's study of state mortality data within the U.S.A. (Burbank, 1972).

In the present study, in addition to an analysis of relationships among cancer sites, *per capita* food consumption figures available for 36 countries were correlated with cancer mortality rates.* The intercorrelations of mortality data and food

* Since this was an initial search for epidemiological clues, the decision was made to use the maximum number of countries for which food consumption figures and age adjusted death rates based on all ages were available. It is recognized that findings may be affected by inclusion of the older age groups in the age adjusted rates as well as the uneven quality of mortality statistics from the various countries.

consumption data were factor analysed in a search for dietary leads in cancer aetiology. While a number of other investigators have reported correlations between food consumption figures and mortality rates for specific cancer sites, this is the first systematic analysis of all sites in relation to available food consumption data.

### MATERIALS AND METHODS

The mortality data analysed were age adjusted cancer death rates per 100,000 for males in 41 countries (Segi, Kurihara and Matsuyama, 1969). (Spain was omitted because of missing data for some sites.) Food consumption figures were from Food Balance Sheets of the Food and Agriculture Organization (FAO) for the period 1964–66 (Food and Agriculture Organization of the United Nations, 1971). The use of earlier data would have been preferable, but this was not possible because of the smaller number of countries for which figures were available. In the food analysis, the first analysis was of major food groups: cereal, starch, sugars, pulses (peas, beans, etc.), vegetables, fruit, meats, eggs, milk, fish and fats (fat oil). An effort was then made to analyse more specific components of the major foods, but very little additional specificity was possible because of the variation from country to country in dietary items. The data were re-analysed, however, based on the following: wheat, rice, potatoes, vegetables, fruit, cattle meat (beef plus veal), pork, poultry, eggs, milk and fish. Appendix Table A-I shows the countries represented in the food analyses.

Because of possible violations of statistical assumptions involved in the calculation of product–moment correlations, rank correlations were calculated. For comparative purposes, the product–moment correlational matrix based on cancer sites was also factor analysed and found to yield the same factors as the rank correlational matrix. Three correlational matrices were obtained, based on the following as variables: (1) cancer sites, (2) cancer sites and major food groups and (3) cancer sites and more specific foods.

Each of the rank correlational matrices was factor analysed by means of a principal components analysis with varimax rotation (Harman, 1960)*. Unities were used as estimates of communality with an eigenvalue of 0·8 as the cut-off for factor extraction and an eigenvalue of 1·0 as the cut-off for factor rotation. As an empirical check on methodology, the mortality data were also factor analysed using the squared multiple correlation of a variable with all remaining variables as the communality estimate (Harman, 1960). The same factors were identified regardless of the method used for estimating communalities.

### RESULTS

*Interrelationships among death rates by cancer site*

Rank correlations and product moment correlations among cancer sites are presented in Table I.

Table II presents the factor analysis results based on rank correlations among cancer death rates for men in 41 countries.

In Table II, factors (which are the hypothetical dimensions accounting for the intercorrelations) are identified by Roman numerals. The coefficients shown for each factor are the factor loadings of the cancer sites on the factor. Sites which have loadings of the same sign (whether positive or negative) on the same factor have mortality rates which vary together (increase or decrease together) across countries. The italicized factor loadings indicate the sites best representing each factor.

A factor loading is the correlation of a variable (site) with an underlying factor or dimension, and a squared factor loading is a coefficient of determination which specifies the amount of variance associated with a site which is accounted for by a particular factor. The sum of squared loadings for a site across *all factors* indicates the variance common (communality [$h^2$]) to that site and the other sites which has been accounted for by the

* The author expresses appreciation to Mrs Karen L. Beckwith for her assistance in obtaining the computer analysis by means of a programme developed by the Biometric Laboratory, University of Miami.

TABLE I.—*Correlations**† *among Age-adjusted Death Rates from Malignant Neoplasms for Selected Sites,* 41 *Countries, Males*‡

| ICD Code | Site | 140–148 | 150 | 151 | 152–153 | 154 | 161 | 162–163 | 177 | 190–191 | 204 |
|---|---|---|---|---|---|---|---|---|---|---|---|
| 140–148 | Buccal cavity and pharynx | | *64* (74) | 10 (–03) | *38* (17) | 20 (07) | *37* (32) | 13 (04) | *32* (07) | *34* (01) | 04 (–04) |
| 150 | Oesophagus | | | *47* (30) | *36* (17) | *31* (10) | *38* (43) | 29 (07) | 30 (19) | 21 (–01) | 08 (01) |
| 151 | Stomach | | | | 05 (00) | *35* (28) | *49* (28) | *36* (27) | 18 (12) | 30 (12) | 12 (22) |
| 152–153 | Intestine, except rectum | | | | | *84* (85) | 13 (14) | *70* (68) | *80* (80) | *63* (60) | *70* (75) |
| 154 | Rectum | | | | | | 18 (17) | *79* (77) | *69* (68) | *61* (49) | *58* (71) |
| 161 | Larynx | | | | | | | *35* (21) | 20 (25) | 23 (15) | 15 (25) |
| 162–163 | Lung, bronchus and trachea | | | | | | | | *59* (54) | *53* (43) | *59* (62) |
| 177 | Prostate | | | | | | | | | *67* (64) | *69* (77) |
| 190–191 | Skin | | | | | | | | | | *64* (64) |
| 204 | Leukaemia and aleukaemia | | | | | | | | | | |

* Decimals omitted.

† Spearman rank *r*'s are shown without parentheses with these significant at the 0·05 level or below italicized; Pearson product moment *r*'s are shown in parentheses.

‡ Rates from Segi, Kurihara and Matsuayma (1969).

TABLE II.—*Rotated Factor Loadings** *Based on Rank Correlations among Death Rates by Cancer Site,* 41 *Countries, Males*

| ICD Code | Site† | Factors I | II | III | $h^2$ |
|---|---|---|---|---|---|
| 140–148 | Buccal cavity and pharynx | 0·14 | −0·05 | *0·94* | 0·91 |
| 150 | Oesophagus | 0·14 | −0·42 | *0·76* | 0·77 |
| 151 | Stomach | 0·13 | −0·91 | 0·04 | 0·84 |
| 152–153 | Intestine, except rectum | *0·90* | 0·07 | 0·31 | 0·91 |
| 154 | Rectum | *0·86* | −0·24 | 0·08 | 0·80 |
| 161 | Larynx | 0·09 | *−0·73* | 0·31 | 0·63 |
| 162–163 | Lung, bronchus and trachea | *0·78* | −0·38 | −0·01 | 0·76 |
| 190–191 | Skin | *0·76* | −0·15 | 0·18 | 0·63 |
| 204 | Leukaemia and aleukaemia | *0·85* | −0·02 | −0·10 | 0·74 |
| 177 | Prostate | *0·85* | −0·03 | 0·25 | 0·78 |
| % of variance | | 42 | 18 | 18 = | 78 |

* Principal components analysis with varimax rotation.

† Italicized factor loadings (the largest for each factor) indicate cancer sites which by inspection are most representative of each factor.

factors. Similarly, the percentage of variance associated with each factor can be obtained from each column of factor loadings.

The factor analysis results in Table II show that, with the ICD codes included in the study, only 3 factors or dimensions were obtained from the statistical associations among cancer sites. These factors accounted for the major part (78%) of the total variance in international cancer death rates for men; the largest factor was Factor I. The $h^2$ column shows that the cancer sites best accounted for by the analysis (having the highest communalities) were buccal cavity and pharynx and intestine; those least well accounted for were larynx and skin.

Factor I, the largest factor, may be considered a westernization or industrialization factor since the sites best representing this factor were those for which death rates tend to be higher in the more westernized countries (Segi *et al.*, 1969). Cancer of the intestine, since it has the largest factor loading, is the site most representative of Factor I. Other cancer sites with high loadings on this factor are rectum, lung, skin, leukaemia and prostate. The high intercorrelations among these cancer sites can be observed in Table I.

Although death rates for the sites represented in Factor I tend to increase or decrease together across countries, there are many common influences affecting the rates. In view of the particular sites loading on Factor I, this factor does not appear to represent a common aetiological dimension. Actually, several aetiological agents may be involved. Causes of lung cancer are cigarette smoking (Cornfield *et al.*, 1959) and air pollution (Raven and Roe, Ed., 1967, p. 181), both of which are more prevalent in the more westernized countries. One cause of skin cancer is sun exposure (Raven and Roe, 1967) which may be greater in the more westernized nations or, alternatively, since groups with less pigmentation are more susceptible to the effects of sunlight, skin cancer may be associated with a skin colour gradient linked to westernization. Radiation, as one causative factor in leukaemia (Raven and Roe, 1967) may be associated internationally with industrialization even though some non-industrialized areas within certain countries have high radiation levels. A dietary pattern, presumed to be related to affluence and westernization, is the most suspect aetiological factor in the development of cancers of the intestine and rectum (Berg, Haenszel and Devesa, 1973; Wynder and Shigematsu, 1967). No explanation, however, can be offered for the association between rates of prostate cancer and westernization.

Factor II is a stomach cancer factor, independent of westernization in which cancer of the larynx was also represented. For cancers of the stomach and of the larynx there are no known common aetiological factors although their representation in the same factor seems reasonable, since the rank correlation of 0·49 between these sites (Table I) was the largest statistical association observed for either of the two sites. The rank correlation between stomach cancer and cancer of the larynx could, of course, be due to the correlation of each of the sites with some other variable (such as social class) not represented in the analysis.

Factor III is represented primarily by cancers of the buccal cavity and pharynx and of the oesophagus; the rank correlation between these sites was 0·64 (Table I). It is to be noted that lung cancer was not represented in Factor III. The implication is that the variance in international rates of lung cancer for males is largely accounted for by the westernization factor (Factor I). Independently of influences associated with westernization (such as cigarette smoking), cancers of the buccal cavity and oesophagus appear to be related. Common aetiological factors may be various types of smoking, alcohol and the chewing of mixtures, tobacco and betel quid (Raven and Roe, 1967).

In general, the use of factor analysis with the mortality data appeared to give a reasonable summary of the statistical associations among cancer sites. The results, however, were interpretable in terms of what is known about cancer causation rather than being suggestive of new hypotheses. Greater specificity in ICD codes could have perhaps yielded more provocative results.

*Interrelationships between* per capita *food consumption figures and death rates by cancer site*

Table III presents rank correlations between international food consumption figures and cancer death rates.

Table IV gives the factor analysis results based on correlations involving major food groups, and Table V gives the parallel results based on more specific

TABLE III.—*Rank Correlations* Between Male Death Rates* (1964–65) *by Cancer Site and* Per Capita *Food Consumption Figures* (1964–66)

| ICD Code: | 140–148 | 150 | 151 | 152–153 | 154 | 161 | 162–163 | 177 | 190–191 | 204 |
|---|---|---|---|---|---|---|---|---|---|---|
| Site: | Buccal cavity | Oesophagus | Stomach | Intestine | Rectum | Larynx | Lung | Prostate | Skin | Leukaemia |
| Major food groups (N = 36) | | | | | | | | | | |
| Cereal | −10 | −11 | 02 | −70 | −60 | 05 | −47 | −68 | −39 | −58 |
| Starch | 14 | 27 | 52 | 30 | 47 | 38 | 36 | 36 | 37 | 20 |
| Sugars | 09 | 11 | −02 | 71 | 59 | −07 | 56 | 67 | 61 | 62 |
| Pulses (peas, beans, etc.) | −03 | −13 | −28 | −59 | −65 | 01 | −65 | −63 | −64 | −66 |
| Vegetables | 17 | 28 | 27 | 35 | 28 | 44 | 24 | 09 | 11 | 30 |
| Fruits | −10 | −24 | −18 | 38 | 25 | 25 | 18 | 38 | 13 | 41 |
| Meats | 28 | 24 | 06 | 89 | 79 | 20 | 72 | 74 | 70 | 62 |
| Eggs | −08 | 12 | 04 | 76 | 72 | 08 | 62 | 50 | 39 | 60 |
| Milk | 00 | 13 | 19 | 68 | 66 | 09 | 61 | 70 | 60 | 73 |
| Fish | 06 | 21 | 08 | −07 | −09 | −21 | −20 | −09 | −31 | 04 |
| Fats (fat oil) | −04 | 02 | 21 | 77 | 80 | 23 | 69 | 73 | 47 | 70 |
| Specific food groups (N = 34) | | | | | | | | | | |
| Wheat | 06 | 14 | 38 | 36 | 40 | 57 | 46 | 32 | 40 | 48 |
| Rice | 10 | 00 | −07 | −72 | −73 | −09 | −59 | −72 | −53 | −67 |
| Potatoes | 00 | 21 | 51 | 58 | 73 | 36 | 60 | 54 | 42 | 54 |
| Cattle meat | 12 | 16 | 01 | 76 | 62 | 16 | 64 | 75 | 69 | 63 |
| Pork | 24 | 21 | 32 | 52 | 69 | 20 | 60 | 41 | 41 | 40 |
| Poultry | 39 | 26 | 09 | 44 | 35 | 48 | 38 | 20 | 40 | 30 |

* Decimals omitted.

TABLE IV.—*Rotated Factor Loadings* Based on Rank Correlations Between Male Death Rates by Cancer Site and* Per Capita *Food Consumption*

36 Countries, major food groups

| Food groups | Factors† I | II | III | IV | V | h² |
|---|---|---|---|---|---|---|
| Cereal | −*0·83* | 0·08 | −0·03 | −0·01 | −0·18 | 0·73 |
| Starch | 0·40 | *0·70* | −0·12 | 0·04 | −0·04 | 0·67 |
| Sugars | *0·85* | −0·08 | −0·19 | 0·07 | −0·26 | 0·84 |
| Pulses (peas, beans, etc.) | −*0·78* | −0·35 | 0·32 | 0·03 | 0·04 | 0·84 |
| Vegetables | 0·11 | 0·17 | *0·82* | 0·17 | 0·09 | 0·75 |
| Fruit | 0·33 | −0·31 | *0·73* | −0·19 | −0·08 | 0·78 |
| Meats | *0·88* | 0·03 | 0·20 | 0·22 | −0·20 | 0·90 |
| Eggs | *0·77* | 0·00 | 0·39 | −0·11 | −0·01 | 0·76 |
| Milk | *0·86* | 0·22 | −0·04 | −0·08 | −0·11 | 0·81 |
| Fish | −0·11 | 0·04 | −0·02 | 0·10 | *0·95* | 0·93 |
| Fats (fat oil) | *0·82* | 0·22 | 0·35 | −0·17 | 0·02 | 0·87 |
| Cancer site | | | | | | |
| Buccal cavity and pharynx | 0·07 | −0·01 | 0·03 | *0·94* | −0·04 | 0·89 |
| Oesophagus | 0·09 | 0·43 | 0·06 | *0·74* | 0·22 | 0·79 |
| Stomach | 0·04 | *0·90* | 0·09 | 0·14 | 0·07 | 0·84 |
| Intestine, except rectum | *0·90* | −0·04 | 0·24 | 0·25 | 0·01 | 0·93 |
| Rectum | *0·81* | 0·31 | 0·18 | 0·08 | 0·00 | 0·79 |
| Larynx | −0·04 | *0·52* | *0·55* | 0·35 | −0·34 | 0·81 |
| Lung, bronchus and trachea | *0·71* | 0·36 | 0·18 | 0·03 | −0·17 | 0·70 |
| Skin | *0·68* | 0·16 | −0·07 | 0·31 | −0·37 | 0·73 |
| Leukaemia and aleukaemia | *0·80* | 0·03 | 0·21 | −0·12 | 0·08 | 0·71 |
| Prostate | *0·84* | 0·08 | 0·06 | 0·18 | −0·05 | 0·75 |
| % of variance | 42 | 11 | 10 | 9 | 7 | 79 |

* Principal components analysis with varimax rotation.

† Italicized factor loadings (the largest for each factor) indicate variables which by inspection are representative of each factor.

TABLE V.—*Rotated Factor Loadings* Based on Rank Correlations Between Male Death Rates by Cancer Site and* Per Capita *Food Consumption*

34 Countries, specific food groups

| Food group | Factors† I | II | III | IV | V | h² |
|---|---|---|---|---|---|---|
| Wheat | −0·33 | 0·23 | *0·76* | 0·09 | −0·13 | 0·76 |
| Rice | *0·86* | −0·17 | −0·04 | −0·23 | 0·07 | 0·83 |
| Potatoes | −0·65 | *0·55* | 0·36 | 0·12 | 0·02 | 0·87 |
| Vegetables | −0·17 | 0·13 | *0·83* | −0·19 | 0·11 | 0·78 |
| Fruit | −0·35 | −0·48 | *0·56* | 0·12 | −0·07 | 0·69 |
| Cattle meat | −*0·78* | −0·22 | 0·13 | −0·15 | −0·14 | 0·72 |
| Pork | −0·59 | 0·43 | 0·03 | −0·16 | −0·22 | 0·61 |
| Poultry | −0·30 | −0·10 | 0·41 | −0·42 | −0·55 | 0·75 |
| Eggs | −*0·79* | 0·05 | 0·22 | 0·01 | −0·16 | 0·70 |
| Milk | −*0·86* | 0·13 | 0·08 | 0·10 | −0·11 | 0·78 |
| Fish | 0·10 | 0·04 | −0·05 | −0·15 | *0·92* | 0·88 |
| Cancer site | | | | | | |
| Buccal cavity and pharynx | −0·05 | −0·02 | 0·06 | −*0·92* | −0·03 | 0·85 |
| Oesophagus | −0·09 | 0·40 | 0·16 | −*0·76* | 0·18 | 0·80 |
| Stomach | −0·07 | *0·78* | 0·34 | −0·18 | 0·09 | 0·77 |
| Intestine, except rectum | −*0·92* | −0·09 | 0·15 | −0·24 | 0·03 | 0·94 |
| Rectum | −*0·84* | 0·30 | 0·14 | −0·08 | 0·04 | 0·82 |
| Larynx | 0·00 | 0·20 | *0·72* | −0·36 | −0·28 | 0·77 |
| Lung, bronchus and trachea | −*0·76* | 0·22 | 0·24 | −0·06 | −0·16 | 0·71 |
| Skin | −*0·72* | −0·01 | 0·07 | −0·28 | −0·24 | 0·66 |
| Leukaemia and aleukaemia | −*0·83* | −0·15 | 0·29 | −0·06 | 0·14 | 0·82 |
| Prostate | −*0·87* | −0·06 | 0·07 | −0·13 | 0·08 | 0·79 |
| % of variance | 38 | 9 | 13 | 10 | 7 | 77 |

* Principal components analysis with varimax rotation.

† Italicized factor loadings (the largest for each factor) indicate variables which by inspection are most representative of each factor.

foods. Because results from the two analyses are quite similar they will be discussed together.

Factor I in both tables is a westernization (standard of living?) factor in which the same cancer sites (intestine, rectum, lung, skin, leukaemia and prostate) appear as were represented in the Factor I of Table II. The foods with factor loadings of the same sign as the cancer sites are those for which consumption increases across countries concomitantly with increases in the death rates for the cancer sites. Such foods among the major food groups (Table IV) are sugars, meats, eggs, milk and fats. These then, are the dietary items characteristic of the affluence of westernized societies. The consumption of cereal and pulses decreases with westernization and is inversely related to the cancer rates represented in Factor I. Rice (Table V), as a specific food in the broader cereal group, is also inversely related to the cancer death rates represented in this factor. On the other hand, the consumption of cattle meat (Table V) increases with increases in the cancer death rates. Cattle meat is the specific meat item, in contrast to pork, poultry and fish, which is most associated with westernization.

The large number of cancer sites and foods represented in Factor I makes interpretation difficult in terms of dietary factors in cancer aetiology. The food correlates of most of the cancer sites represented in the westernization factor may not be of aetiological significance in that the correlational pattern which produced Factor I may be due to correlations of both the cancer sites and the foods with other variables. For cancers of the intestine and rectum, the sites most likely to involve dietary factors, the results in Tables IV and V are not inconsistent with speculation about the role of dietary fat (Wynder and Shigematsu, 1967) or animal protein (Gregor, Toman and Prusova, 1969) in the development of cancer of the large bowel. A case control study of colorectal cancer (Berg *et al.*, in the press) has specifically implicated meat, particularly cattle meat or beef, as being of aetiological importance. Support for the case control results was suggested by an earlier factor analysis based on *selected* cancer sites and by diet survey data considered in relation to U.S. incidence and mortality data (Howell, unpublished report).

Factor II in both tables is a stomach cancer factor in which the major food group represented (Table IV) is starch and the more specific food is potatoes (Table V). This factor is compatible with the suggestion that a diet high in carbohydrates may be related to gastric cancer (Wynder, Graham and Eisenberg, 1966). The factor also has support from case control results which showed higher potato consumption among gastric cancer cases than among controls (Graham, Schotz and Martino, 1972). It is to be noted from Table V that potato consumption, although represented in the stomach cancer factor, also showed a fairly strong relationship (factor loading of — 0·65) to westernization (Factor I). This suggests that although potato consumption may be relatively high in the westernized countries, the total dietary pattern is of importance since gastric cancer rates in these countries tend to be low (Segi *et al.*, 1969).

From Table IV. it can be observed that cancer of the larynx had a much lower factor loading on Factor II than was true in Table II when the analysis was limited to the mortality rates. In the food analyses (Tables IV and V), cancer of the larynx split from stomach cancer to be represented in Factor III, which was associated with the consumption of vegetables, fruit and wheat. Factor III in these analyses is an unexpected finding. While the highest food correlate of cancer of the larynx is wheat, the rank correlation of this site with vegetables and fruit is not higher than those with some of the other foods (Table III). The representation of vegetables and fruit in this factor can occur, statistically, as a result of the total pattern of intercorrelations, including those (which are not presented) among food consumption figures. Since no explanation can be offered for this factor, any search for aetiological meaning for Factor III might be limited to be largest food correlate of larynx cancer, that is,wheat consumption.

Factor IV in Tables IV and V is a buccal-oesophageal cancer factor which was not associated with any of the foods included in the analysis. This is consistent with what is known about the development of cancers of these sites in that suspect aetiological agents (Raven and Roe, 1967) do not currently include foods.

Factor V (Tables IV and V) is a factor representing fish consumption which is not associated with cancer rates for any site. Although fish consumption appears unrelated to the cancer death rates, it should be pointed out that the available *per capita* food consumption information did not permit study of the methods of fish preparation such as smoking and salting.

Of the foods analysed, only pork and poultry were *not* clearly associated with a particular factor, but were represented in several factors. One of these (Factor I) showed some, but not a strong relationship, between pork consumption and westernization (Table V).

### DISCUSSION

The westernization factor and two other factors accounted for the major part of the variance in international cancer death rates for men. The second factor represented stomach cancer with which cancer of the larynx was associated, perhaps because of correlations of the death rates for these sites with other variables not included in the analysis. The third factor was a buccal–oesophageal cancer factor which was interpreted as reflecting common aetiological agents in the development of carcinomata of these sites.

A search for dietary factors in cancer aetiology was made through factor analysis of *per capita* food consumption data in conjunction with cancer mortality rates. A number of food correlates of the westernization factor were found, one of which (cattle meat) was interpreted as having significance in the development of colorectal cancer in view of similar findings from a case control study (Berg *et al.*, in the press). The stomach cancer factor was associated with starch consumption, specifically potatoes, which also is supported by case control results (Graham *et al.*, 1972). Surprisingly, cancer of the larynx was associated with the consumption of vegetables, fruit and wheat; no explanation of this finding could be offered but the association with wheat consumption may be worth further exploration. Death rates from buccal–oesophageal cancers were found *not* to be associated with dietary factors.

Although this study has not been primarily a methodological one, it is of some interest that factor analysis used with the kinds of data analysed here did provide a meaningful summary of intercorrelations and clarified the observed statistical associations. With greater specificity in basic data than was possible here and with other types of data, not necessarily death rates or food consumption figures, factor analysis could prove to be a useful and perhaps powerful epidemiological tool.

APPENDIX TABLE A–I.—*Countries for Which* Per Capita *Food Consumption Figures Were Available*, 1964–66

| | |
|---|---|
| South Africa | Germany, Fed. Rep. |
| Canada | Greece |
| Chile | Hungary |
| El Salvador | Ireland |
| U.S.A., white | Italy |
| Mexico | Netherlands |
| Panama | Norway |
| Puerto Rico | Poland |
| Venezuela | Portugal |
| Taiwan | Sweden |
| Hong Kong | Switzerland |
| Japan | Yugoslavia |
| Philippines | Czechoslovakia |
| Thailand | United Kingdom |
| Austria | Australia |
| Belgium | New Zealand |
| Denmark | Israel* |
| France | Finland* |

* Not included in the analysis of specific foods.

### REFERENCES

BERG, J. W., HAENSZEL, W. & DEVESA, S. S. (1973) Epidemiology of Gastrointestinal Cancer. In *Proc. Seventh natn. Cancer Congress.* Philadelphia: Lippincott. p. 459.

BURBANK, F. (1972) A Sequential Space–Time Cluster Analysis of Cancer Mortality in the United States: Etiologic Implications. *Am. J. Epidemiol.*, **95**, 393.

CORNFIELD, J., HAENSZEL, W., HAMMOND, E. C., LILIENFELD, A. H., SHIMKIN, M. B. & WYNDER, E. L. (1959) Smoking and Lung Cancer: Recent Evidence and a Discussion of some Questions. *J. natn. Cancer Inst.*, **22**, 173.

FOOD AND AGRICULTURE ORGANIZATION OF THE UNITED NATIONS (1971) *Food Balance Sheets, 1964–66 Average.* Rome: Food and Agriculture Organization.

GRAHAM, S., SCHOTZ, W. & MARTINO, P. (1972) Alimentary Factors in the Epidemiology of Gastric Cancer. *Cancer, N.Y.*, **30**, 927.

GREGOR, O., TOMAN, R. & PRUSOVA, F. (1969) Gastrointestinal Cancer and Nutrition. *Gut*, **10**, 1031.

HARMAN, H. H. (1960) *Modern Factor Analysis.* Chicago: University of Chicago Press. p. 89.

RAVEN, R. W. & ROE, F. J. C. (Eds.) (1967) *The Prevention of Cancer.* New York: Appleton-Century-Crofts. p. 91, 212, 343.

SEGI, M., FUJISAKU, S., KURIHARA, M., NARAI, Y. & SASAJIMA, K. (1960) Age-adjusted Death Rates for Malignant Neoplasms in Some Selected sites in 23 Countries in 1954–55 and Their Geographical Correlation. *Tohoku J. exp. Med.*, **72**, 91.

SEGI, M. & KURIHARA, M. (1972) *Cancer Mortality for Selected Sites in 24 Countries, No.* 6 (1966–67). Tokyo: Japan Cancer Society.

SEGI, M., KURIHARA, M. & MATSUYAMA, T. (1969) *Cancer Mortality for Selected Sites in 24 Countries, No.* 5 (1964–65). Sendai: Department of Public Health, Tohoku University School of Medicine.

WYNDER, E., GRAHAM, S. & EISENBERG, H. (1966) Conference on the Etiology of Cancer of the Gastrointestinal Tract. Report of the Research Committee, World Health Organization on Gastroenterology, New York. New York, June 10–11, 1965. *Cancer, N.Y.*, **19**, 1564.

WYNDER, E. L., HYAMS, L. & SHIGEMATSU, T. (1967) Correlations of International Cancer Death Rates. *Cancer, N.Y.*, **20**, 113.

WYNDER, E. L. & SHIGEMATSU, T. (1967) Environmental Factors of Cancer of the Colon and the Rectum. *Cancer, N.Y.*, **20**, 1520.